# Ureteral Hematoma: A Case Report on Complications During Anticoagulant Therapy

**DOI:** 10.7759/cureus.28666

**Published:** 2022-09-01

**Authors:** Coulibaly Moussa, Keita Ansoumane Hawa, Meriam Benzalim, ALJ Soumaya

**Affiliations:** 1 Radiology, Centre Hospitalier Universitaire (CHU) Mohammed VI, Marrakech, MAR

**Keywords:** back pain, hematuria, anticoagulant, hematoma, ureteral

## Abstract

Ureteral hematoma is a rare complication occurring during anticoagulant therapy, with fewer than 10 cases reported in the literature. Bleeding complications are underestimated. They affect about 10% of patients treated with long-term anti-vitamin K (AVK). The appearance of macroscopic hematuria may indicate the presence of underlying organic damage. Clinically, ureteral hematoma is manifested by lumbar or abdominal pain often associated with macroscopic hematuria. Imaging plays a major role in its diagnosis. Clinical and radiological evolution is always rapidly favorable after the correction of coagulation disorders and the immediate discontinuation of anticoagulant treatment. We report in this work the case of a patient in her 50s who presented a ureteral hematoma during her anticoagulant treatment.

## Introduction

Ureteral hematoma is a rare complication occurring during anticoagulant therapy. Fewer than 10 cases have been reported in the literature. The appearance of macroscopic hematuria may indicate the presence of underlying organic damage, or it may be an exceptional manifestation directly related to the hemorrhagic effect of the treatment [1]. Clinically, ureteral hematoma is manifested by back or abdominal pain associated with macroscopic hematuria [2]; imaging plays a major role in its diagnosis. We report in this work the case of a patient in her 50s who presented with a ureteral hematoma during her anticoagulant treatment.

## Case presentation

A 51-year-old patient was seen in the emergency room for macroscopic hematuria associated with bilateral lumbar pain, predominantly on the left side. The patient had a history of hypercoagulability discovered during an episode of deep vein thrombosis, for which long-term treatment with anti-vitamin K (AVK) was instituted.

On clinical examination, the patient was conscious and apyretic with tenderness of the lumbar fossa without palpable mass or bladder globe. The rest of the examination did not reveal any notable particularity. On biological examination, the international normalized ratio (INR) was elevated to 12, and the prothrombin ratio (PR) to 8%. The cytobacteriological examination of the urine was sterile for culture. The blood count showed no anemia, thrombocytopenia, or hyperleukocytosis. Renal function and C-reactive protein (CRP) were normal.

**Figure 1:**
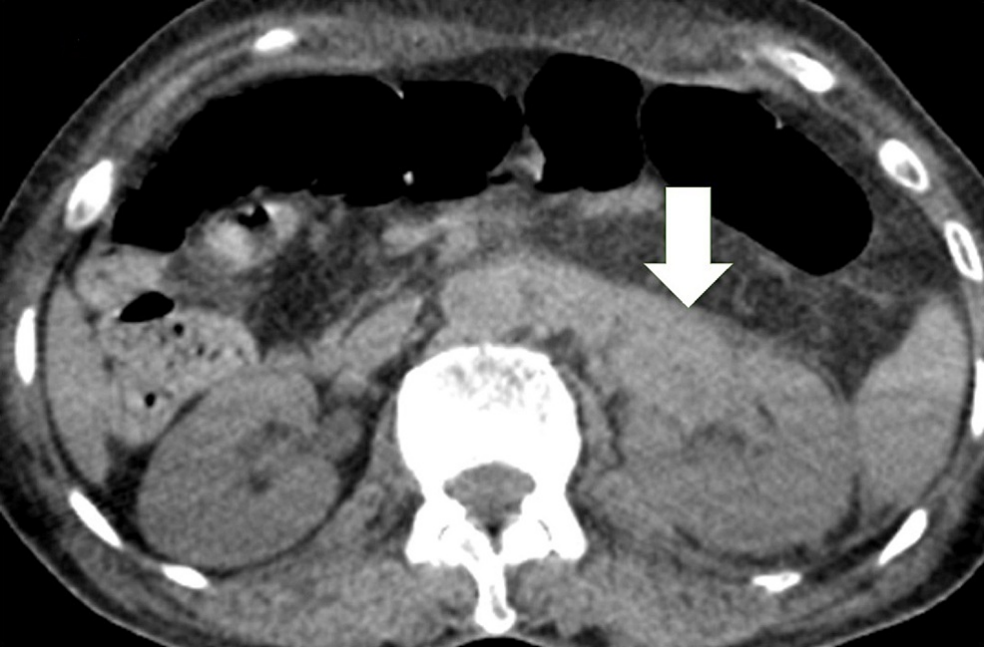
Uro-scanner without injection of contrast medium showed a spontaneously hyperdense left ureteral parietal thickening (white arrow).

**Figure 2:**
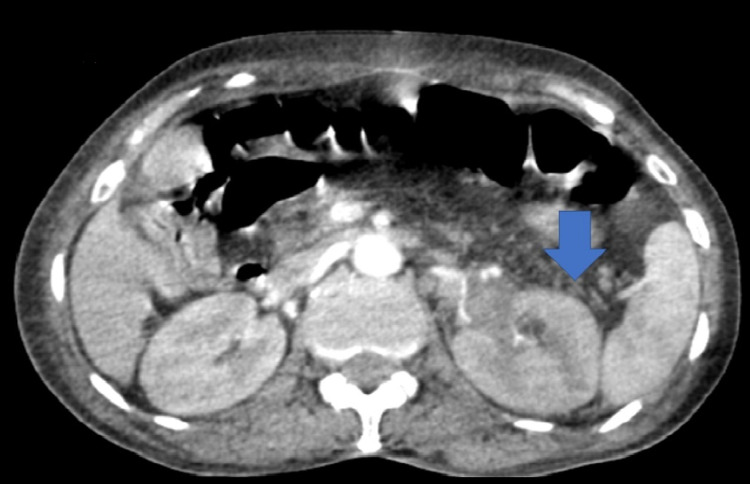
Uro-scanner after injection of contrast medium at arterial time with a demonstration of a left ureteral parietal thickening, spontaneously hyperdense, and infiltration of the perirenal fat (blue arrow).

**Figure 3:**
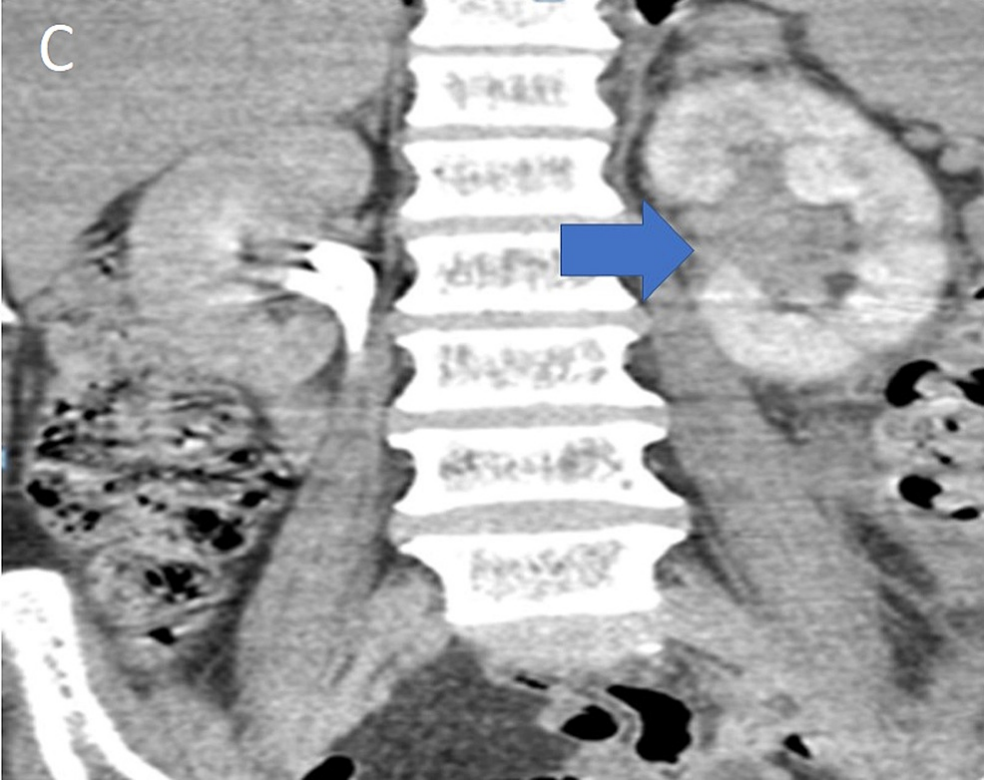
Uro-CT scan with injection of contrast medium at excretory time: coronal section illustrating the dilatation of the pyelocalic cavities (blue arrow) with a delay in excretion.

**Figure 4:**
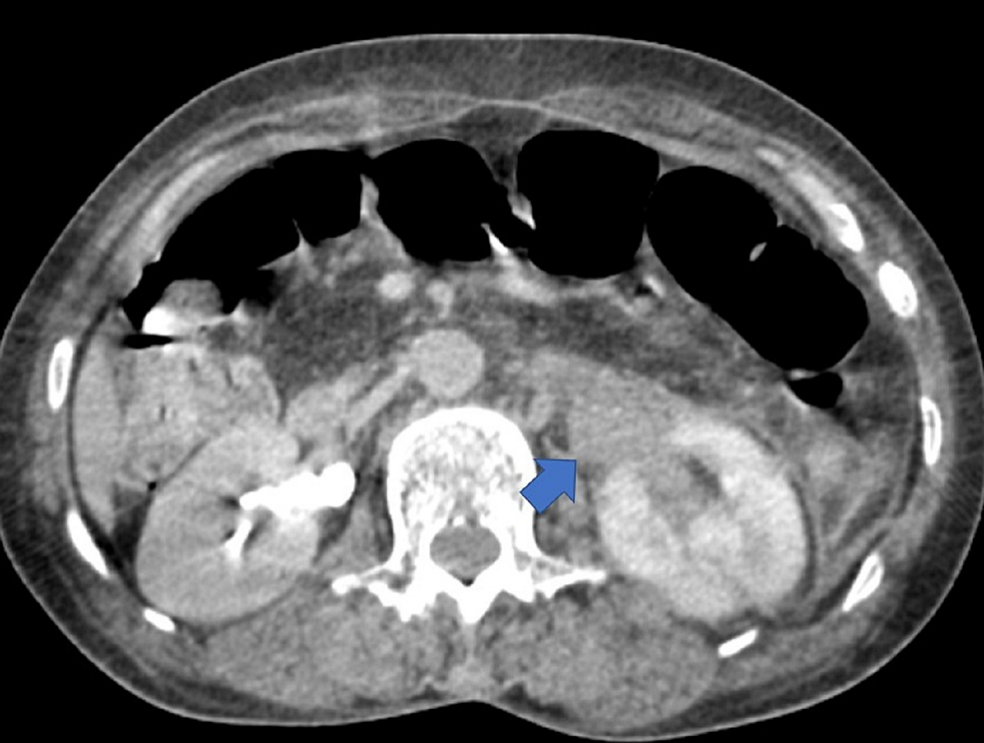
Uro-CT scan with injection of contrast medium at excretory time: axial section illustrating dilatation of the pyelocalic cavities upstream of a ureteral hematoma (blue arrow) with a delay in excretion.

On imaging, the ultrasound of the urinary system was unremarkable; on the non-injected sections, the computed tomography (CT) showed a thickened left ureteral wall that was spontaneously hyperdense, with no visible stone image in the excretory tract (Figure 1). On the injected series, there was a proximal left ureteral and pylecal dilatation associated with a parietal thickening of the renal pelvis and infiltration of the homolateral perirenal fat (Figure 2). At excretory time, there was a delay in excretion in the left kidney (Figures 3, 4).

Cystoscopy, ureteroscopy, and urine cytology studies were negative, with no evidence of underlying urothelial tumor. Given the context of anti-vitamin K (AVK) overdose and the isolated nature of the macroscopic hematuria with a negative urological etiology, the diagnosis of left ureteral hematoma due to anti-vitamin K (AVK) overdose was retained.

**Figure 5:**
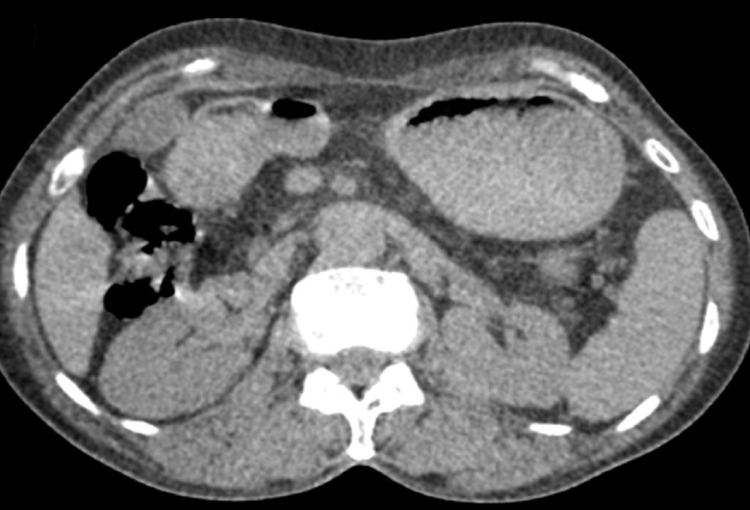
Control CT scan without injection of contrast medium (axial section): complete disappearance of the left ureteral hematoma and the homolateral perirenal infiltration without dilatation of the pyelocalic cavities.

The treatment was conservative, based on immediate discontinuation of anti-vitamin K (AVK) drugs with relay by heparin therapy. The evolution was favorable after three weeks with the disappearance of macroscopic hematuria and lumbar pain. In the fourth week of treatment, a control uro-scan was performed, revealing the disappearance of the ureteral parietal thickening and of the infiltration of the perirenal fat (Figure 5).

## Discussion

Ureteral hematoma is a rare complication occurring during anticoagulant therapy, with less than 10 cases reported in the literature. Hemorrhagic complications are poorly described but probably underestimated [3]. They affect about 10% of patients treated with long-term anti-vitamin K (AVK) therapy [4]. The pathophysiology of this condition remains unexplained, and it can be deduced from the analogy with segmental hematoma of the intestine that hematic infiltration occurs in the connective tissue and muscularis propria and then spreads by contiguity due to ureteral peristalsis. The most frequent clinical symptomatology is hematuria, which represents 20%-40% of hemorrhagic events. It usually requires a global etiological workup in search of an organic pathology [4]. This hematuria may progress to a hematoma. Hematomas, whether pyelic, parietal, or ureteral, are generally located at the submucosal level [3]. They are symptomatic in the majority of cases and are clinically manifested by lumbar or abdominal pain associated with macroscopic hematuria. They may occur in the absence of biological overdose [2].

In imaging, computed tomography is currently the first-line examination because of its better resolution, and its performance is not reduced by macroscopic hematuria [5]. It allows the demonstration of a parietal pyelic or ureteral hematoma. It is necessary to perform it in the presence of any hematuria occurring in a patient on oral anticoagulant treatment to look for an organic etiology, which is encountered in more than three-quarters of cases. It shows a unilateral or bilateral parietal thickening, regular or not, of the pyelon and proximal ureter. This thickening may be spontaneously hyperdense with little or no enhancement after injection of contrast medium. It may be associated with the infiltration of the surrounding fat, particularly in the perirenal, periureteral, and perirenal fascia. Dilatation of the pyelocaliceal cavities may be observed, or even an excretory delay, depending on the degree of obstruction [3]. Intravenous urography (IVU), formerly used as a reference examination, has lost its value in the diagnosis of ureteral hematoma due to advances in imaging techniques. The main radiological differential diagnoses are nonspecific pyeloureteritis evolving in an infectious context, idiopathic retroperitoneal fibrosis that does not resolve in a few weeks, and urothelial tumor in case of irregular thickening of the pyloric wall [1,5]. A regression of the symptomatology after 3-4 weeks after stopping the treatment is an argument in favor of the diagnosis. The literature reports that clinical and radiological evolution is always rapidly favorable, even in cases of acute obstructive renal failure, after the correction of coagulation disorders and the immediate discontinuation of anticoagulant treatment [1].

## Conclusions

Ureteral hematoma is a rare complication occurring under anticoagulant therapy that is not well known and probably underestimated. Computed tomography is currently the examination of choice because it allows the evaluation of the extent of the hematoma and its impact on the excretory cavities. It also allows for differential diagnosis and posttreatment monitoring. Clinical and radiological evolution is always rapidly favorable after the correction of coagulation disorders and the immediate discontinuation of anticoagulant treatment.

## References

[1] Cabaniols L, Laffargue G, Gres P, Guiter J, Thuret R (2008). [Anticoagulant therapy complicated by ureteric haematoma: a case report]. Prog Urol.

[2] Velut JG, Bagnères D, Portier F, Bagatini S, Demoux AL, Chaumoître K, Frances Y (2000). [Ureteral hematoma complicated by anticoagulant treatment: a case report and review of the literature] [Article in French]. Rev Med Interne.

[3] Kolko A, Kiselman R, Russ G, Bacques O, Kleinknecht D (1993). Acute renal failure due to bilateral ureteral hematomas complicating anticoagulant therapy. Nephron.

[4] Van Savage JG, Fried FA (1995). Anticoagulant associated hematuria: a prospective study. J Urol.

[5] Descotes JL, Hubert J, Lemaitre L (2003). [Urology imaging: contribution of imaging in upper urinary tract tumors]. Prog Urol.

